# Digital Literacy at an Urban Cancer Center: Implications for Technology Use and Vulnerable Patients

**DOI:** 10.1200/CCI.21.00039

**Published:** 2021-08-24

**Authors:** Amy E. Leader, Lisa M. Capparella, Lauren B. Waldman, Rebecca B. Cammy, Alison R. Petok, Rebecca Dean, Ayako Shimada, Liana Yocavitch, Kristin L. Rising, Gregory D. Garber, Brooke Worster, Adam P. Dicker

**Affiliations:** ^1^ Sidney Kimmel Cancer Center, Thomas Jefferson University, Philadelphia, PA; ^2^ School of Social Policy and Practice, University of Pennsylvania, Philadelphia, PA

## Abstract

**PURPOSE:**

eHealth literacy, or the ability to seek, find, understand, and appraise health information from electronic sources, has become increasingly relevant in the era of COVID-19, when so many aspects of patient care became dependent on technology. We aimed to understand eHealth literacy among a diverse sample of patients with cancer and discuss ways for health systems and cancer centers to ensure that all patients have access to high-quality care.

**METHODS:**

A cross-sectional survey of patients with cancer and caregivers was conducted at an NCI-designated cancer center to assess access to the Internet, smartphone ownership, use of mobile apps, willingness to engage remotely with the health care team, and use of the patient portal. Descriptive statistics and bivariate analyses were used to assess frequencies and significant differences between variables.

**RESULTS:**

Of 363 participants, 55% (n = 201) were female, 71% (n = 241) identified as non-Hispanic White, and 29% (n = 85) reported that their highest level of education was a high school diploma. Most (90%, n = 323) reported having access to the Internet and most (82%, n = 283) reported owning a smartphone. Younger patients or those with a college degree were significantly more likely to own a smartphone, access health information online, know how to download an app on their own, have an interest in communicating with their health care team remotely, or have an account on the electronic patient portal.

**CONCLUSION:**

As cancer centers increasingly engage patients through electronic and mobile applications, patients with low or limited digital literacy may be excluded, exacerbating current cancer health disparities. Patient-, provider- and system-level technology barriers must be understood and mitigated.

## BACKGROUND

The COVID-19 pandemic is an unprecedented and historic event that caused a fundamental shift in how health care is delivered to and received by patients. The rapid uptake of telemedicine and patient portals,^1,2^ both for continued health care and access to COVID testing and vaccines, is convenient and efficient for providers but requires resources and digital health literacy from patients. Research is just beginning to understand how reliance on these technologies is affecting vulnerable and under-resourced patients.^3,4^ Here, we describe an assessment at our cancer center of our patients' and caregivers' ability to access and use common technology platforms and describe the implications of our findings in the context of health care in a postpandemic world.

**CONTEXT**

**Key Objective**
The COVID-19 pandemic has caused increased reliance on technology to access cancer care. It is important to understand the distribution of eHealth literacy, or the ability to seek, find, or understand health information from electronic sources, in a cancer patient population to know where disparities may exist. We surveyed 363 patients with cancer and caregivers to document their eHealth literacy, as well as predictors of eHealth literacy.
**Knowledge Generated**
We found that older patients, those with a lower educational level, and those from a minority race or ethnicity had the lowest levels of eHealth literacy. They were the least likely to have access to or use technology in their everyday life and use it in managing their cancer care.
**Relevance**
Cancer centers must be cognizant of these disparities when using technology with patients, to not widen cancer health disparities.

Universal health literacy has been identified as a public health goal for the 21st century.^5^ In today's digital society, one of the most relevant aspects of health literacy is electronic health literacy, or eHealth literacy. eHealth literacy has been defined as the ability to seek, find, understand, and appraise health information from electronic sources and apply the knowledge gained to addressing or solving a health problem.^6^ Although sufficient health literacy has been associated with positive health outcomes, the impact of eHealth literacy on health outcomes has been less explored,^7^ but is assumed to be equally important. The pathway from eHealth literacy to health outcomes may be through patient engagement,^8^ such that patients with higher eHealth literacy can more effectively self-assess symptoms, communicate with providers, digest electronic information about their health, and manage biometric measures and medications.

A cancer diagnosis is often accompanied by an onslaught of new information and new health care teams. A recent study found that more than 90% of patients with cancer turn to the Internet to access information about cancer before speaking to a health care professional.^9^ Research shows that although the interest in using technology to manage cancer care is high, the actual adoption of such technology among patients with cancer is much lower and disparities in use exist.^10,11^ Disparities in eHealth literacy and use are most frequently seen among older patients, those who have lower socioeconomic status, and those who identify with a racial or ethnic minority group.^12^ Finally, patients with lower health literacy report lower levels of shared decision making^13,14^; by extension, it can be assumed that patients with lower eHealth literacy would also report lower levels of shared decision making, although this has yet to be explored.

The purpose of this study was to assess differences in patient access to and use of digital technology and electronic health information in their daily lives and in relation to their cancer care. The study was stimulated by an interest in using digital health to help our patients. As we began to consider remote monitoring for patient-reported outcomes, fundamental questions regarding digital literacy were raised. We surveyed a diverse sample of patients and caregivers who received care from a large, urban NCI-designated cancer center. We expected to see the greatest disparities in eHealth literacy among our older patients with cancer, those who have fewer years of education, and those who identify with a minority racial or ethnic group. The results of the study can inform best practices for ensuring that all patients with cancer have access to information and their health care providers, as well as informing future interventions to reduce the digital literacy divide among medically underserved patients.

## METHODS

### Participants

As patients checked in for scheduled appointments in either Medical Oncology or Radiation Oncology, they were given a paper-based survey to complete either on their own or with assistance if they had difficulties or questions. A smaller sample of patients completed the survey on an electronic tablet in our Center Support and Welcome Center and were offered assistance or a paper survey if they preferred. All patients with cancer and caregivers were eligible to participate in the survey if they were older than age 18 years and able to read and speak English. All participants provided verbal consent before beginning the survey. Survey data were collected anonymously. Participants were not compensated for their time. We received expedited approval from our institution's institutional review board to conduct the survey.

### Survey

The 24-item survey (Data Supplement) was created by the research team to meet the needs of the cancer center and was divided into four content areas: access and use of the Internet for health information; smartphone ownership; use of mobile health apps and wearable technology; and use of technology to interact with health care professionals. For the first content area, we asked questions about where patients and caregivers typically access the Internet, the type of device they typically use to access the Internet, whether they have ever visited a web site to learn about cancer, and whether they have ever visited our cancer center's web site to learn about resources available to them. For the second content area, we asked participants whether they have a cell phone or smartphone, the brand of the phone, how much time they typically spend on their phone, and what they do on their phone. For the third content area, we asked participants how many apps are currently on their phone or tablet, if any of the apps are health-related, if they can find and download an app on their own, if they have ever used an app to help with a decision related to their cancer care, and if they routinely use any wearable technology. For the fourth content area, we asked whether participants would be comfortable communicating with their provider over their phone in a telemedicine visit and whether they use our cancer center's electronic portal to manage their cancer care. Finally, we collected demographic information from each participant to assess whether they were a patient or a caregiver, their age, sex, race or ethnicity, zip code, and educational achievement. The survey took about 10 minutes to complete.

### Data Analysis

Descriptive statistics were computed for all variables. Some demographic variables were collapsed based on sample distribution and recoded for bivariate analyses. Age was categorized as below age 60, between age 60-69, and older than age 70 years. Race and ethnicity were categorized as non-Hispanic White or minority race or ethnicity (included non-Hispanic Black, Hispanic, and Asian participants). Differences in technology use by sex, ages, race or ethnicities, and education levels were tested by chi-square tests. Statistical significance was defined as *P* < .05 on a two-tailed distribution. To evaluate the associations between (1) find and download an app unassisted, (2) willing to do telehealth, and (3) use of electronic patient portal and participants' characteristics (age, education level, and race), three separate logistic regression model analyses were conducted. For characteristics with more than two categories, type-III *P* values representing the overall association of the characteristic with the digital literacy measure were calculated as were pairwise comparisons with a reference category. The significance level of all tests was set a priori to the .05 level. All analyses were performed with SAS 9.4. Missing data, which were determined to be minimal and at random, were excluded from analyses on a question-by-question basis.

## RESULTS

### Description of Participants

Four hundred and fifty (n = 450) patients and caregivers were approached to complete the survey and 363 participants completed the survey. Three hundred and forty-seven (n = 347, 96%) were completed on paper and 16 (4%) were completed on tablet computer. Three hundred forty-six (n = 346, 96%) were patients, 13 (4%) were caregivers, and four were unknown. Almost half of the participants (n = 151, 42%) were younger than age 60 years, whereas an additional one quarter (n = 84, 23%) were older than age 70 years. Fifty-five percent of participants (n = 201) were female, whereas 45% of participants (n = 160) were male. The majority of participants were non-Hispanic White (n = 241, 66%), whereas 23% of participants (n = 85) were non-Hispanic Black. Educational attainment was divided among participants: 27% (n = 99) had a high school diploma, 17% (n = 60) had a trade or an associate degree, 23% (n = 85) obtained a bachelor's degree, and 15% (n = 53) had an advanced degree. Characteristics of participants are displayed in Table 1.

**TABLE 1.**
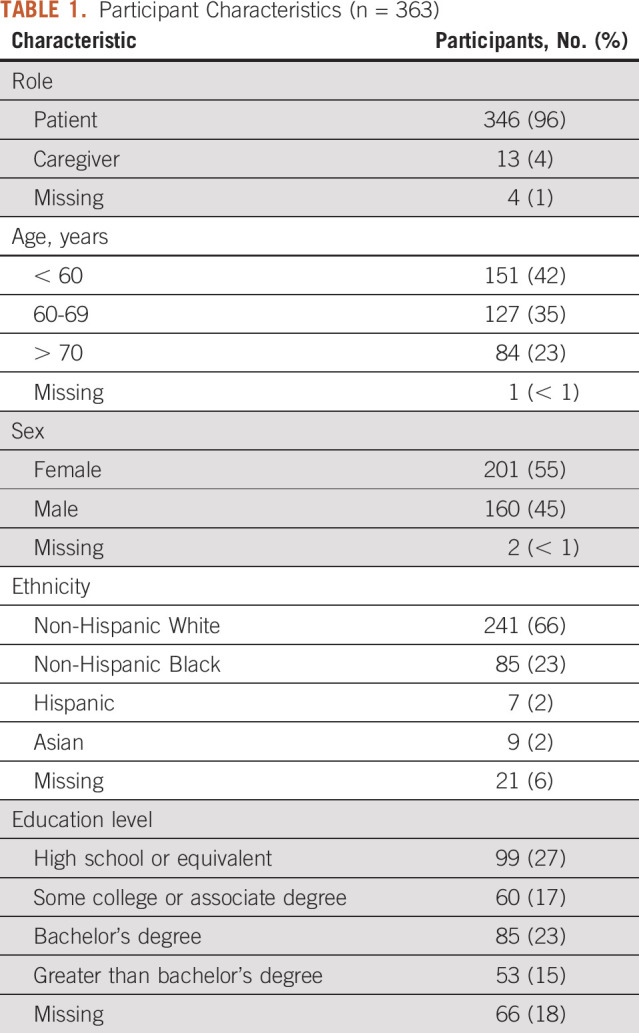
Participant Characteristics (n = 363)

### Access and Use of the Internet for Health Information

Thirty-seven participants (10%) reported that they do not use the Internet at all. Among those who do, patients and caregivers predominantly accessed the Internet at home (n = 309, 86%), followed by at work (n = 77, 20%) and at a public place (n = 22, 6%). Participants reported that they accessed the Internet in multiple ways, using computers (n = 248, 70%), cell phones (n = 191, 54%), and tablets (n = 125, 35%). Most participants (n = 252, 69%) reported that they had visited a web site to learn more about their cancer. These data are presented in Table 2.

**TABLE 2.**
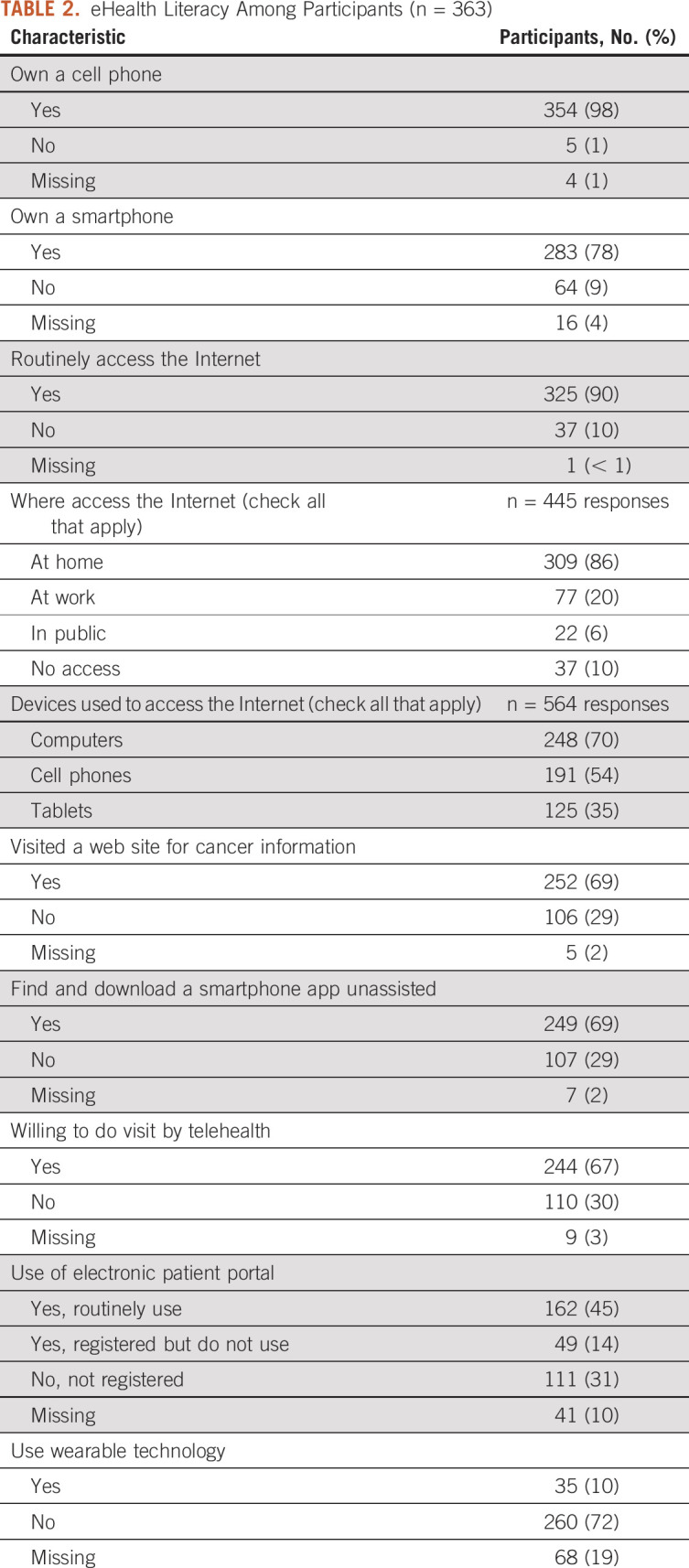
eHealth Literacy Among Participants (n = 363)

### Smartphone Ownership

Nearly all participants had a cell phone (98%, n = 354); for 283 (80%) of those participants, their phone is a smartphone. Among the 283 participants who own a smartphone, 55% (n = 160) reported spending less than 2 hours per day on their phone. Thirty percent reported using their phones 2-4 hours per day (n = 86) and 15% (n = 44) reported using their phones for more than 4 hours per day. Participants reported using their smartphones primarily for communication (n = 269, 95%), accessing the Internet (n = 201, 71%), and navigating to places (n = 170, 60%).

### Use of Mobile Health Apps and Wearable Technology

Only about 70% of participants (n = 249) reported that they can find and download them on their own. Most (62%, n = 225) did not have a health-related app on their phone or tablet. Only 10% (n = 35) reported using wearable technology, including brands like Fitbit and Apple Watch. These data are presented in Table 2.

### Using Technology to Interact with Health Care Professionals

Sixty-seven percent of participants (n = 244) said that they would feel comfortable communicating with their doctor or nurse using a smartphone or tablet (ie, a telehealth visit). Just under half of the participants (45%, n = 162) said they use the cancer center's patient portal fairly often, 14% (n = 49) said they signed up for access to the patient portal but do not use it often, and 31% (n = 111) said they do not use the patient portal. These data are presented in Table 2.

### Differences in Technology Use by Age, Educational Attainment, and Race or Ethnicity

There were significant differences in the use of technology by age. The most notable were that older participants were less likely to access the Internet, own a smartphone, download an app by themselves, or have an interest in communicating electronically with providers. Almost all participants, across all educational levels, reported owning a cell phone (98.6%, n = 355). However, there were significant differences in the use of technology by educational attainment. The largest disparities were that those with only a high school education were less likely to access the Internet, visit a web site for health purposes, be able to download an app by themselves, have an interest in communicating electronically with their providers, or have any wearable technology. Finally, there were significant differences by race or ethnicity of the respondent. Participants from minority races and ethnicities were significantly less likely to routinely access the Internet, visit a web site to look for cancer information, be able to find and download an app on their own, and use the patient portal. There were no reported differences in technology use by sex. All these data can be found in Table 3.

**TABLE 3.**
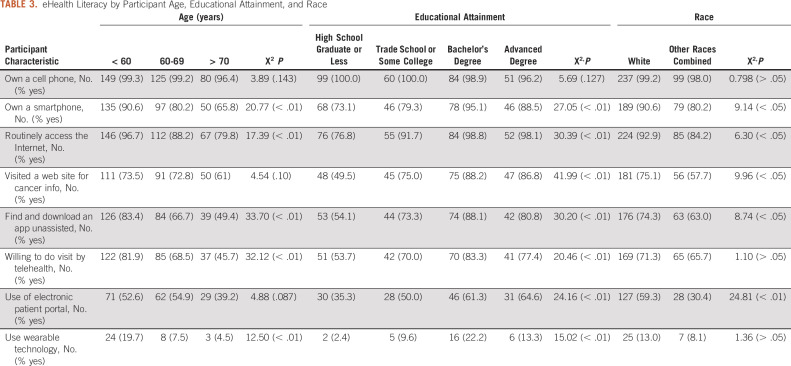
eHealth Literacy by Participant Age, Educational Attainment, and Race

### Prediction Models for Important eHealth Skills

We aimed to understand which factors would be predictive for three key eHealth literacy skills: finding and downloading an app unassisted, willingness to do a telehealth appointment, and using a patient portal. Age and educational attainment were significantly predictive across all three models, with those who were younger or had more education being more likely to use digital technology. In one model, using a patient portal, those who were not White were significantly less likely to use a patient portal for their health care needs. The results of the three models are found in Table 4.

**TABLE 4.**
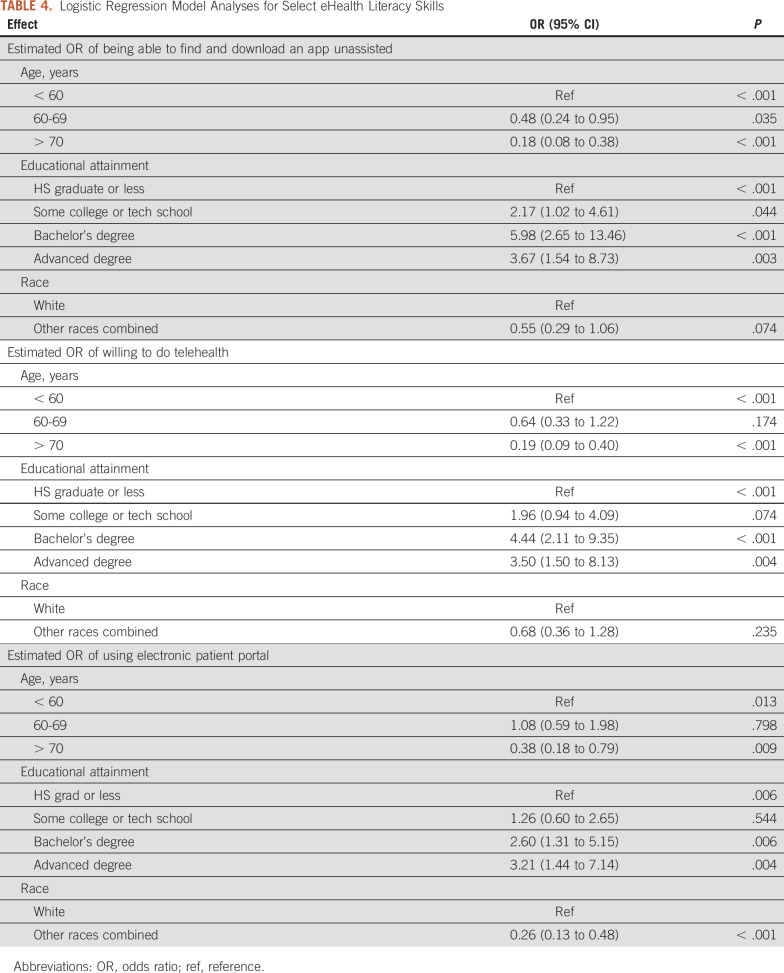
Logistic Regression Model Analyses for Select eHealth Literacy Skills

## DISCUSSION

Our study aimed to document eHealth literacy among a diverse sample of patients with cancer and caregivers at an urban, academically based cancer center. We found that although access to the Internet and smartphone use was relatively high among our patients and caregivers, disparities in access and use of the technology exist among our most vulnerable patients with cancer, most notably those who are older and of lower educational attainment. Although this has been documented previously,^15,16^ it remains worrisome as it is evidence that we are expanding, rather than closing, the digital divide among patients and caregivers who need access to information and technology to manage their disease and interact with their care team.

Given these findings, the question of how to provide the same level of access to health information and health care to all patients, regardless of eHealth literacy level, is an important one. Patient navigators, which are deployed by many health systems to provide an extra level of patient support, may be able to overcome some of the technical barriers of cancer care.^17^ Whether it is teaching them how to download and use a health app to track their cancer care or create an account on the patient portal, patient navigators or support personnel may be one answer to ensuring equal access for all. Our cancer center is currently offering classes to teach patients and caregivers how to sign up and access their electronic health records. We created a telehealth task force to walk patients through the process of conducting a telehealth visit. Other suggestions include linking patients to community resources that provide free or low-cost home or publicly available Internet access.^18^ Recently, many of these recommendations were included in an 18-point Digital Universal Precautions for health care organizations that want to make digital health accessible and meaningful for all patients, regardless of digital health literacy.^3^

Because of the COVID-19 pandemic, providers and health systems were forced to reimagine health care, much of it transitioning to virtual platforms. In our study, we found that patients who are older and have lower educational attainment are less willing to participate in telehealth visits; patients of racial and ethnic minority heritages are less likely to use our patient portal to manage their care. Knowing these patients with cancer are already at increased risk for poor cancer outcomes and now are at increased risk of poor outcomes from the pandemic,^19^ leaving these patients behind with our continued use of technology could have a disastrous impact. To ensure that the expansion of telemedicine does not exacerbate health disparities, four key actions were recently proposed: (1) proactively exploring disparities in telemedicine access; (2) developing solutions to mitigate barriers; (3) removing health system–created barriers to access; and (4) advocating for policies and infrastructure that facilitate equitable access.^20^ This encompasses the need for health systems to create and use technology that patients want to adopt to manage their health care needs.^21^

The research described here can be used as a starting point toward developing a tool to identify those with low eHealth literacy, to be able to deploy resources and support to those who need assistance in navigating health care in a digital world. Although a few digital literacy screening tools exist or are in development,^22-25^ none have the ability to screen for skills that we feel are essential in this postpandemic era such as downloading an app, conducting a telehealth visit, or logging into a patient portal. While we acknowledge that screening tools should not replace efforts to provide universal access to literacy-appropriate health care,^26^ identifying and assisting those at greatest risk of not being able to access care remains an important aspect of supportive cancer care.

There are limitations to our study. While comprising a large sample of patients with cancer and caregivers, it was a convenience sample of patients and the results may not be generalizable to the overall cancer patient population. We limited our sample to English-speaking patients and were not able to capture eHealth and digital literacy disparities that are known to exist in the Hispanic and Latino population.^27^ These data are self-reported and do not explore why some participants are not using certain technologies. The survey that participants completed was created by the research team to provide data on questions and concerns that were most pressing to the cancer center and deviated from using known scales of eHealth literacy that, although validated,^22-25^ were not as relevant to our needs.

Going forward, as health systems and cancer centers unveil new programs and options for patients, they must be cognizant that not all patients have access to these technologies nor the capabilities or knowledge to use them to their full capacity. Unfortunately, there are few evidence-based approaches to increasing eHealth literacy in socially disadvantaged groups.^28^ As more appointments are scheduled and occur online and through telehealth, as more cancer centers rely on patient-generated health data to track symptoms and evaluate quality of care,^29^ and as more information is posted exclusively on web sites and through social media platforms, there is a segment of the patient population that will not be privy to these services. Leadership must remain cognizant of disparities in eHealth literacy and ensure that until every patient can fully participate, a low-tech option must be available. Directing all patient traffic to a web site, a portal, or an app may be easier and more cost effective for the health system but it does so at a cost to vulnerable patients.
